# RETRACTION: Artificial Intelligence for Emergency Medical Care

**DOI:** 10.1002/hcs2.70007

**Published:** 2025-04-04

## Abstract

**RETRACTION**: S. Rajput, P. K. Sharma, and R. Malviya, “Artificial Intelligence for Emergency Medical Care,” *Health Care Science* (Early View), https://doi.org/10.1002/hcs2.72.

The above article, published online on 13 October 2023 in Wiley Online Library (wileyonlinelibrary.com), has been retracted by agreement between the journal Editor‐in‐Chief, Zongjiu Zhang; Tsinghua University Press; and John Wiley & Sons Ltd. The retraction has been agreed due to unattributed overlap between this article and a previously published article [1]. The corresponding author R. Malviya disagrees with the retraction. S. Rajput and P. K. Sharma were informed of the decision to retract but remained unresponsive.
